# Acknowledgement to Reviewers of *Veterinary Sciences* in 2015

**DOI:** 10.3390/vetsci3010005

**Published:** 2016-01-27

**Authors:** Veterinary Sciences Editorial Office

**Affiliations:** MDPI AG, Klybeckstrasse 64, CH-4057 Basel, Switzerland; vetsci@mdpi.com

The editors of *Veterinary Sciences* would like to express their sincere gratitude to the following reviewers for assessing manuscripts in 2015.

We greatly appreciate the contribution of expert reviewers, which is crucial to the journal’s editorial decision-making process. Several steps have been taken in 2015 to thank and acknowledge reviewers. Good, timely reviews are rewarded with a discount off their next MDPI publication. By creating an account on the submission system, reviewers can access details of their past reviews, see the comments of other reviewers, and download a letter of acknowledgement for their records. We are grateful to our reviewers for the contribution they make.
Andre, CatherineGroner, BerndPetrovski, KiroArthur, HelenGünther, SebastianPinchuk, LesyaBaeumer, WolfgangHalsey, Charles H. C.Poglayen, GiovanniBarnhart, KirstinHendricks, Joan C.Poli, AlessandroBeineke, AndreasHogan, JosephPoroyko, ValeriyBennett, PeterHolmes, MarkPourquier, PhilippeBerry, Donagh PHorvath, GyörgyQueiroga, Felisbina Luisa Bertoni, Prof. GiuseppeInobe, ManabuRebhun, RobertBloom, PaulJayne, DavidRodriguez, CarlosBoothe, Dawn MertonKania-Korwel, IzabelaRosol, Thomas J.Bradford, BarryKnapp, DeborahSakai, HirokiBriner, WayneKrimer, Paula M.Scherk, MargieBryant, JulietLakritz, JeffreySehgal, InderCannon, Claire MLiao, Albert T.Sierra, EvaCannon, Claire M.Lopez Gatius, FernandoSong, Chang-SeonChaves, RaquelMackin, AndrewSteffen, DavidCiccarelli, MicheleMani, RinoshStein, TimConstantino-Casas, FernandoManiscalco, LorellaSu, HuaDe Rensis, FabioMarkey, BryanSuter, StevenDobschuetz, Sophie vonMaterniak, MagdalenaTardy, FlorenceDuato, Esperanza Gomez-Lucia Matsuda, HiroshiTervaert, Jan Willem CohenDuesbury, NickMcCleary-Wheeler, Angela L.Thamm, Douglas H.Elder, John H.Meinecke-Tillmann, SabineVerma, AshutoshFan, Timothy M.Milovancev, MilanVillinger, FrancoisFox, LarryOh, S. Paulvon Euler, HenrikGallagher, Alexander E.Ohta, HiroshiWalter, IngridGeyer, JoachimPage, RodneyWendt, Anna-MariaGoga, Beniamino Terzo CenciPassler, ThomasWestman, MarkGrassi, Felice RobertoPelletier, GuillaumeWhite, StephenGrasso, FeliciaPereira, Marcos NevesYoung, Howard

